# University Students’ Engagement in Mobile Learning

**DOI:** 10.3390/ejihpe13010016

**Published:** 2023-01-14

**Authors:** Reham Salhab, Wajeeh Daher

**Affiliations:** 1 Faculty of Educational Sciences, An-Najah National University, Nablus P400, Palestine; 2 Department of Mathematics, Al-Qasemi Academic College of Education, Baqa-El-Gharbia 3010000, Israel

**Keywords:** mobile learning, mobile technology, student engagement, university students

## Abstract

The implementation of mobile learning seems to be an emerging topic in many educational institutions. As recently noticed, mobile technology has employed wireless technologies to communicate, think, learn, and share in order to spread and exchange information. Therefore, using mobile technologies in learning and teaching can create a positive environment in higher education. Hence, the purpose of this study is to evaluate mobile learning engagement among educational technology students. Data from three focus group discussions and 15 semi-structured interviews with students who experienced mobile learning were gathered using a qualitative approach design. A total of seventeen basic themes and four organizing themes were extracted, where the researchers categories of engagement, i.e., social engagement, cognitive engagement, emotional engagement, and behavioral engagement. In the present research, the findings indicate that social engagement themes included social–mobile interaction, building community, developing relationships, and competition. The cognitive engagement themes included attention, cognitive and meta-cognitive strategies, immersion, and cognitive curiosity. Emotional engagement themes included excitement and enjoyment, instructor comforting students, motivation, and emotional safety. Behavioral engagement themes included effort and time on task, attendance, participation, and positive conduct.

## 1. Introduction

Ubiquitous and pervasive emerging technologies, such as computing technology, have paved the way to harnessing the power of technology tools to improve the learning environment and strengthen the education system [1,2,3,4]. Actually, they have made information more accessible and promoted quality and effective learning, as well as disseminated knowledge more easily [5,6,7,8]. Thus, learning through digital media enables students to seamlessly utilize various kinds of functional objects anytime and anywhere through a network of connections which personalize, accelerate, and empower students to take control of their learning [9]. Due to these emerging pedagogical applications, universities have recently started utilizing mobile learning in education [6,10]. Because mobile learning has a favorable impact on students and promotes learning engagement, it can enhance the learning environment in higher education [10]. Moreover, it facilitates accessing students’ learning activities, which will promote interaction, communication, and assignment completion [11].

Mobile applications are easily accessible; students can access digital content in a very personalized manner with their connected digital devices. Consequently, this would enhance their learning outcomes by engaging them more [11,12,13]. Engaged learners can boost their motivation, attention, and focus, as well as enhance their interaction and cooperation [14].

### 1.1. Significance of the Study

One of the main challenges that educators are confronted with is student engagement, which has to be resolved if educators want to enhance the quality of education while utilizing mobile learning [15,16]. Learners’ engagement plays a crucial role in any learning experience in higher education institutions. Moreover, instructional designers and educators can integrate engagement elements into a mobile learning environment. This study attempts to study students’ engagement in the mobile context, where previous studies have shown that this context can enhance students’ achievements, interest, and a sense of community in the mobile learning process [9,17].

### 1.2. Statement of the Problem

Given the rapid global increase in mobile device users, over 6.64 billion people are using smartphones worldwide. In other words, 83.72% of the world’s population owns a smartphone, and 7.26 billion people own mobile devices, making up 91.54% [5]. The usage of mobile devices for learning has increasingly become popular [18]. Obviously, mobile learning is one of the mobile technologies that have shown many promising benefits in higher education fields. It provides a flexible educational environment [19]. Recently, several studies have shown that mobile learning increases interaction and engagement in learning [20,21].

After reviewing the literature, it is clear that there is a lack of intensive research and studies looking at the impact of implementing mobile learning to enhance students’ engagement [5,16,22,23,24], particularly in Palestinian schools [25]. Hence, this study will add to previous studies by exploring the impact of m-learning on student engagement in higher education institutions. Consequently, this study aims to investigate university students’ engagement in m-learning in an educational technology course. To accomplish this, the researchers depended on Bowden et al. [26] to study the engagement processes deductively, but inductive reasoning also accompanied the analysis, where through this reasoning we arrived at the specific themes associated with engagement in the mobile environment. Depending on Bowden et al., we considered engagement to have four components: the social, the cognitive, the affective, and the behavioral. To further affiliate themes with each component, we depended on inductive reasoning.

### 1.3. Literature Review

#### 1.3.1. Mobile Learning (m-Learning)

Mobile technology is one of the innovative communication technological tools that has exponentially become omnipresent in our daily life [27]. As a result of recent advancements in mobile telecommunications and computing technologies such as mobile phones, tablets, smart watches, PDS, and digital audio players, m-learning flourished through using mobile technology for teaching and learning [28,29].

Several definitions have been given to m-learning. Alkhalaf et al. [30] defined it as “the use of small and hand-held portable wireless devices, such as mobile phones, digital assistants, smartphones, and small personal computers to achieve flexibility and interaction in the teaching and learning processes anytime and anywhere”.

Recently, Traxler [31] defined m-learning as “a type of learning that assists learners in acquiring knowledge, attitudes, skills, and processes with connectedness and mobility”. Mobility is a unique feature of m-learning that provides flexibility in time, place, and speed [32]; students can learn anywhere and anytime. However, various challenges may appear, such as security, privacy, safety, and communication issues [33]. Using multimedia, such as text, images, audio, and videos, is another essential feature of *m-learning*.

Researchers noticed that higher education institutions have started implementing m-learning in teaching and learning [34,35]. This implementation would request studying it using theoretical frameworks. The present research intends to do so.

#### 1.3.2. Engagement in Education

Learning engagement is a priority and has critical impact on any learning environment [36]. Even though the phrase ‘student engagement’ is a dynamic, diverse, and complicated meta-construct [37], many researchers have described and defined it as students making attempts to participate during the learning process [38,39]. This effort can be observed by any number of behavioral, cognitive, social, and emotional indicators [40,41].

There is an ongoing investigation of the engagement processes in education [42,43,44], where some talk about three components of engagement (emotional, cognitive, and behavioral) [45,46], while others talk about four components, and others add social engagement [42,47]. According to research by Henri et al. [48], there are four dimensions of student engagement: emotions, cognitions, behaviors, and social aspects. Both institutional and individual factors can affect these dimensions. However, researchers are not conclusive about how to measure this multidimensional construct [49]. It is critical and well acknowledged that learning engagement increases students’ chances of success [18].

To shed light on the significance of student engagement, Pham and Chen [5] mention that engagement is a pathway to facilitating success. Similarly, Dunn and Kennedy [15] argue that students’ level of engagement in educational settings is directly related to their advancement in the classroom. According to them, nothing may help students to improve academically more than taking an active role in the classroom. An association between engagement and achievement is also stated since it influences retention. Furthermore, learning engagement is demonstrated by how students participate in discussions and collaborate on projects to build knowledge [21,22,23].

#### 1.3.3. Engagement in Mobile Learning

Mobile learning has the potential for meaningful engagement in the learning process. Previous research has shown that the use of smartphones motivates university students to actively participate in online courses, even if they are shy to ask questions in face-to-face classes due to social stigma [50]. Additionally, students who use smartphones can communicate online with their peers, as they can easily ask their friends or lecturers about educational issues through online platforms that support social interaction [51,52,53]. Researchers who have a particular interest in m-learning have looked into how applications such as Udemy, LinkedIn Learning, Coursera, edX, and Skillshare can increase learners’ engagement. Their findings suggest that mobile applications can increase engagement among college students [16]. Heflin et al. [54] explore the effectiveness of m-learning, where students use a collaborative learning environment to facilitate engagement and critical thinking. Results revealed positive responses from students when utilizing mobile collaborative learning, with increased learner engagement during class. M-learning activities are found to be relatively helpful in boosting positive effects on motivational and emotional engagement among college students when the influence of game mobile applications has been examined on motivational and inspirational engagement [51].

### 1.4. Theoretical Framework

Researchers defined students’ engagement as commitment, effort, involvement, and participation [55]. They argued that learner engagement can be viewed as a broad phrase that encompasses learning both inside and outside the official academic contexts. In this research context, the main interest is m-learning engagement. The most frequently used questionnaire was the National Survey of Student Engagement (NSSE) [56]. This institution-level questionnaire assesses the quality of a student’s college experience and addresses behavioral, cognitive, and emotional dimensions of engagement. This engagement survey is still heavily influenced by conventional settings [48].

This study has drawn upon the literature within higher education and shows that students’ engagement is conceptualized through four dimensions [26].

Hence, engagement in learning activities and discussions is referred to as “behavioral engagement” [57], whereas “emotional engagement” is related to the summative levels of emotions that students experience and can be demonstrated by enthusiasm, boredom, happiness, and pride [58]. Social engagement considers the bonds of belonging among students, instructors, and their peers [59], and cognitive engagement embodies setting goals and applying thinking processes [57]. Researchers have stressed that *m-learning* engages students by enhancing the knowledge shared through interaction among students, peers, and instructors within and outside of the classroom [60]. M-learning adaptation in the educational system of higher education institutions is required to assure the equity and quality of education as it is a tool with considerable power that can provide new possibilities for education and learning [61]. In this context, the study searches for answers to the following questions:

### 1.5. Research Questions

What characterizes students’ engagement in the mobile phone environment?

Four questions follow the main question:

What characterizes the social engagement of students in the mobile phone environment?

What characterizes the cognitive engagement of students in the mobile phone environment?

What characterizes the affective engagement of students in the mobile phone environment?

What characterizes the behavioral engagement of students in the mobile phone environment?

## 2. Methodology

### 2.1. Research Settings and Participants

The qualitative approach was used in this study because it investigates insights and thoughts rather than statistical analysis [62]. A phenomenological approach was conducted to determine student engagement in m-learning from students’ perspectives. Phenomenology focuses on describing and interpreting how individuals experience a specific phenomenon in the context of their environment [63]. Correspondingly, the study aimed to comprehend the phenomenon of students’ engagement in their m-learning experiences. Twenty-five third- and fourth-year students from the College of Arts and Educational Sciences at the Technology Education Department of Palestine Technical University Khadoorie (PTUK), the majority of whom were females, participated in the study. They were enrolled in an Educational Technology course, which is a compulsory course for Technology Education students.

### 2.2. Research Context

This study was carried out in the Educational Technology course at Khadoorie University (PTUK). It aimed to achieve six goals, including (i) designing educational materials such as posters and brochures about subjects in the technology school curriculum, (ii) creating educational videos of lessons in the technology curriculum, (iii) utilizing Google applications to design a site that integrates Google tools, (iv) creating educational blogs, (v) creating Google sites, and (vi) creating online quizzes and surveys using educational websites and applications such as Wordwall, Kahoot, Padlet, and Edmodo. In addition, the course utilized short content videos, discussions, forums, and assignments.

The course design utilized ADDIE (Analysis, Design, Development, Implementation, and Evaluation) as an instructional design model. The course was redesigned with the same objectives, which included activities such as wikis, blogs, discussion forums, micro learning videos, open educational resources (OERs), assignments, self-assessment, pop quizzes, and group work. Mobile-supported collaborative learning projects, such as creating interactive presentations of technology curriculum lessons, and individual activities such as educational video creation, assignment submission, discussion forms, and online exams, were conducted. This course was delivered using a mobile Moodle application already adapted and carried out by smartphones and other mobile devices.

### 2.3. Data Collection Tools

Two collection tools used were focus group discussions and post-course interviews. Focus group discussions provide extensive and diverse information where participants listen to each other’s responses which simulates more insights to develop ideas clearly and to clarify misconceptions [64]. A focus group is defined as a structured discussion between a group of people where a moderator is involved who addresses issues to be discussed and ensures all members participate and the conversation stays on topic, controlling any overly dominant members [65]. Focus groups with four to seven participants are typical for collecting data on the experiences, interactions, and perspectives of the participants [66]. The session lasted approximately 120 min for the three groups. Semi-structured interviews provide in-depth data and resolve seemingly conflicting thoughts, since the researcher has the opportunity to inquire about the apparent conflicts [67].

A purposive sampling procedure was used to choose the participants for the focus group discussions and the interviews. The participants were selected according to their willingness to participate in the study and were interviewed after obtaining their agreement to participate [68]. The selection criteria for this phenomenological study were first identifying participants who experienced the phenomenon of m-learning in the course [69]. Online meetings, focus group discussions, and interviews were conducted using Zoom, a virtual video conference tool, following the guidelines for online data collection suggested by [70].

Semi-structured interviews were conducted at the end of the course to gather rich data on participants’ learning experiences and engagement [71]. Four months after the course started, a forty-minute interview was conducted. It consisted of open-ended questions about the participants’ overall learning experiences, any technical issues they encountered while using the Moodle mobile application, and their engagement processes. The focus group and interview combination aided data interpretation and strengthens the veracity of the findings [72].

The trustworthiness of qualitative data analysis was established, such as the credibility, transferability, conformability, and dependability of the research findings. Trustworthiness is a degree of confidence in data, in the preciseness and consistency of data analysis, and in the disclosure of methods of analysis used to guarantee the quality of research [73]. To ensure credibility, different research instruments, such as interviews and focus group discussions, were used for data triangulation. The researchers adopted a code–recode approach for reliability; the same data were coded twice, with a two-week gestation period between each coding. A comparison was made between the two coding results to determine whether they were similar or different. Additionally, stepwise replication was adopted; the two researchers analyzed the same data separately and compared the results. For transferability, purposive sampling was based on specific criteria. Conformability was satisfied by the researchers by documenting the procedures and drawing conclusions based on participant narratives and language rather than any researcher biases. Moreover, double checking of the data was performed throughout the study.

### 2.4. Ethical Consideration

The study was conducted ethically; participants were informed about the confidentiality of their identity and that the interview data would only be used for research purposes. Additionally, they were informed that participation was not compulsory, and they could withdraw at any time. To protect participants’ privacy, their personal identifications have not been declared in the study and their contact information has been stored in a secure and locked computer [74].

### 2.5. Data Analysis Tools

Transcripts from focus groups and interviews were analyzed using inductive and deductive content analysis. The content analysis included an abstraction process for the chunks of data so that the researchers could answer the study questions using categories and themes using inductive reasoning [75]. Inductive analysis was used to generate categories that emerged from participants’ responses. In the deductive analysis, an engagement framework and its four dimensions social, behavioral, emotional, and cognitive were adopted [26]. The constant comparison analysis method was used to analyze data as well. Since there were multiple focus groups in this study, constant comparison analysis was suitable for data analysis, and allowed the researcher to assess across-group saturation in particular and saturation in general.

Table 1 shows themes for the different types of engagement, where these themes were arrived at inductively. The goal of the table is to show the themes and codes related to each motivational category. This will help other researchers to perform similar research for other learning environments. These themes can traced in the findings, where these themes are accompanied by interpretation and quotations from the students.

## 3. Results

To answer the study questions (what characterizes students’ engagement in the mobile phone environment?) and the related questions, the researchers analyzed the data to identify themes for the four types of engagement. Below, we describe each emerging category and its themes.

### 3.1. Social Engagement

Students described how they participated in mobile social activities and maintained a social network during m-learning. Five subthemes were found: building community, mobile social interaction, developing relationship, sense of belonging, and competition.

#### 3.1.1. Building Community

Social forums in mobile applications that have been integrated into Moodle make it easier for participants to engage in tasks that require some level of interpersonal connection. Students who use m-learning interact with their classmates’ work. A student said, “Having to comment on a peer’s post on Padlet, Edmodo made me realize that I am not alone; I am part of this class”. The previous saying illustrates that m-learning helps students to stay connected and assist one another with mobile content learning.

#### 3.1.2. Mobile Social Interaction

Students have been mostly involved in interactions with content, interactions with peers, and instructors while using mobile devices, and interactions with interfaces that facilitate mobile engagement and the sharing of information. A student commented, “When using a mobile device and interacting with course content and classmates was totally different from online learning, we have more control and learning is more flexible”. This shows that using the Moodle mobile application promotes social mobile interaction since it offers interactive content.

#### 3.1.3. Developing Relationships

This refers to students’ relationships or social connections. Learning flexibility improved connections between students and gave them a chance to form new bonds. A student stated, “Mobile learning helped me make new friends; we were helping each other, supporting each other, and getting to know each other very well, I was able to socialize with my classmates many times and by different means in each lecture when we used our mobile phones”. The students’ responses indicate that m-learning can enhance students’ relationship building.

#### 3.1.4. Sense of Belonging

Students who used the Moodle mobile application experienced a sense of belonging to their group in this class. Increased social integration that fosters a sense of belonging may result from social interaction and ongoing conversation with classmates. Students, who were interviewed, indicated that they were extremely excited when they were asked to use their mobile phones to open the Moodle application and start working with counterparts and instructors and helping other classmates. A student commented, “I never felt alone; I was able to stay connected with peers who I take classes with daily. M-learning enabled me to know other students’ personal characteristics, which made us closer”.

#### 3.1.5. Competition

Students could indulge their competitive nature thanks to the use of mobile apps such as Wordwall, Socrative, and Kahoot. Gamification applications provide players with a reward system. Students who answer correctly feel that they are the best. Since everyone wants to win, they compete to get the best results. They try to win by learning and understanding the concepts before playing the game. A student stated, “I like the ranking system when we use Kahoot; it motivates me to win the game, it is fun and challenging because I can compete with classmates, and learning while playing, it creates a stress-free learning environment”. This means that students like to perform better and better for every quiz so that they can be at the top of the scoreboard when they use mobile applications such as Kahoot.

### 3.2. Cognitive Engagement

Based on the FGD and interview data, cognitive engagement is measured among students. Four subthemes were found: attention, cognitive and meta-cognitive strategies, immersion, and cognitive curiosity. M-learning supports the transformation of student cognitive engagement in multiple segmenting materials into “bite-size” installments by way of short micro-lecture videos.

#### 3.2.1. Attention

Students in FGD and interviews mentioned that m-learning makes them more attentive and focused on tasks. Most students assert that using mobile phones grabbed their attention. According to the interviews, many students became more attentive to mobile activities since mobile applications contain a variety of activities and interactive instructional videos. A student mentioned, “By using my mobile phone, I would have more opportunities to share my thoughts instantly, which require more attention from my side to seek answers to my questions from my classmates, to share ideas that help me learn and think critically, and to put my best efforts into coming up with a quality answer”. This saying suggests that m-learning can enhance cognitive engagement by making students more attentive to concepts since many puzzle applications such as Wordwall and Kahoot make learning fun.

#### 3.2.2. Cognitive and Meta-Cognitive Strategies

Another aspect of m-learning is improving cognitive strategies such as problem-solving skills and using multimedia for memorization. A student commented, “Using micro-content and mini-videos on the Moodle app in this course makes me more attentive since it is easy to create short posts with easy mobile access”. Moreover, m-learning was found to improve meta-cognitive strategies such as problem-solving, evaluation, and the monitoring of students’ thinking. Many students indicated that their technical and academic skills for searching have improved as m-learning supports their problem-solving skills.

Mobile learning arose to improve meta-cognitive methods such as identifying the learner’s style and needs, planning tasks, assembling and organizing a workspace, monitoring errors, gauging task success, and evaluating the learning approach. One student said, “I learned by playing games and found it the most appropriate learning strategy to fit my needs; learning by using the mobile device helped me identify my learning style”. Students were able to recall information quickly, and they enjoyed the micro-learning and H5PL films, which shows that cognitive engagement promotes cognitive and meta-cognitive techniques.

#### 3.2.3. Immersion

Students described a state whereby they were so engaged in mobile activities that nothing else seemed to matter. A student mentioned, “The lecture went by so quickly that I did not notice the time when I was playing the Wordwall puzzle!” This assumes that students are engaged cognitively and feel immersed while using m-learning activities.

#### 3.2.4. Cognitive Curiosity

As the learner explores and browses for concepts, m-learning fosters cognitive curiosity, according to many students. “Using our mobile devices helps us look for information instantly and more flexibly”, a student assured; this demonstrates that students become more curious when they use m-learning.

### 3.3. Emotional Engagement

Based on the FGD and interview data, emotional engagement is measured among students. Four subthemes were found: excitement and enjoyment, instructor comforts students, motivation, and emotional safety.

#### 3.3.1. Excitement and Enjoyment

The majority of participants in focus groups and interviews explicitly acknowledged the positive emotions they felt while using m-learning. The following factors were cited by students as reasons why m-learning improved their emotional engagement: M-learning during this course helped students create a sense of excitement and enjoyment. A student said, “My mobile phone is part of my soul, I felt relaxed when the instructor asked as to log into Moodle, it is just a great sensation that makes me happy”. This saying asserts that m-learning facilitates enjoyment and excitement resulting from the nature of mobile devices being part of a student’s life and they see them as full of joy and fun.

#### 3.3.2. Instructor Comforts Students

When using m-learning among students and their peers, instructors find ways to connect with students and promote connection. They play a crucial role in boosting students’ enthusiasm and supporting them emotionally. The feeling of connectedness was an indicator of this dimension. The interviewees emphasized the significance of the instructor comforting students; they also say that they appreciated the instructor’s attempt to use these mobile devices in an academic setting and think the instructor was approachable. One student said, “We felt connected with our instructor all the time, who helped us to ensure we had great support to enable us to succeed”. This indicates that while students are on their phones, the instructor’s consolation is critical for emotional support, encouragement, and feedback.

#### 3.3.3. Motivation

Many students said they felt motivated when using m-learning because of its interactivity, accessibility to a wide range of information sources, and ease of use. “You cannot hide during m-learning, but you do in traditional environments”, a student said. Many students were motivated to show up and be seen by their classmates, and be more visible. This encourages you to perform well in a group or even individually; you do not want to feel like someone who cannot solve problems. This shows that motivation is one of the indicators of emotional engagement; designing interactive activities motivates students to learn and perform better.

#### 3.3.4. Emotional Safety

Students reported that they feel more confident and less shy in an m-learning environment where they can express their ideas and thoughts. The following are some statements from students: “I felt that my voice did not seem good enough to be heard in class; by using mobile devices I am more confident and feel less shy to express myself and share my ideas”. It seems that emotional safety is another aspect of emotional engagement during m-learning; students are less confident in answering during traditional teaching. This reveals that emotional engagement, as described by participants, has four indicators: the first is enjoyment and excitement; the second is the instructor’s comforting of students; the third is motivation; and the fourth is emotional safety.

### 3.4. Behavioral Engagement

Researchers measured behavioral engagement based on focus group discussions and interviews. Four sub-themes were extracted: effort and stay on task, attendance, participation, and positive conduct.

#### 3.4.1. Effort and Stay on Task

Mobile learning induced behavioral engagement by helping students to put more effort in and stay on task. “Using mobile devices in class was a new experience; it was a challenge because I am a recognized student, and I have to work hard to stay on track; I dedicated special effort to using connected m-learning resources, and I would always make some extra effort to get a little above average”, said one student. This confirms that m-learning boosts student behavioral engagement by helping them put more effort in and stay focused.

#### 3.4.2. Attendance

M-learning encourages students to attend class regularly, participate more in class and outside of class settings, and follow positive conduct. This kind of engagement emerged from FGD interviews.

#### 3.4.3. Participation

Students are taking part in m-learning activities extensively. Most students participated more while learning on their devices than through traditional teaching. A student mentioned, “I am diabetic, I missed many classes due to my illness, and m-learning helped me participate while I am home!” This demonstrates that because m-learning is flexible and mobile, it encourages student participation.

#### 3.4.4. Positive Conduct

A student stated, “Many students showed that they respected each other’s thoughts and decisions; students also demonstrated an increased participation rate by attending this class regularly and posting in-class and out-of-class activities”. This indicates that students engage behaviorally by respecting each other’s thoughts and ideas.

## 4. Discussion

Students’ engagement in the digital classroom has attracted the attention of researchers for its impact on their learning [76,77,78,79,80]. The present study has been conducted to investigate students’ engagement in m-learning. In order to accomplish this, the Moodle mobile application was implemented in an educational technology course. A framework for engaging with dimensions was adopted [26]. The research results indicated that students were likely to be engaged socially, cognitively, emotionally, and behaviorally when using mobile devices to access learning content; these findings are in line with [81,82,83]. The reasons behind this are that they clearly understood concepts, enjoyed learning, put in the effort, and interacted with peers.

Based on the findings of these results and their merging and categorization procedures, 17 basic themes were extracted from focus groups. The extracted basic themes were placed into four main themes, including dimensions of mobile engagement in these courses.

The research results indicated that m-learning can enhance social engagement. M-learning is characterized by affording wider and more diverse social interactions than online learning since it facilitates participation in mobile social activities and enhances relationships. In addition, learners are empowered to take complete control of their mobile social interactions. Mobile social interaction is where learners are connected and constantly interacting with content, platforms, peers, and instructors. Because of this, m-learning can help students to create more social interaction than with online or network-based learning. This result is consistent with prior studies [84].

Students confirmed that m-learning offers many options for personalized learning and different learning styles such as games, micro-learning videos, discussions, images, concept maps, and interactive videos. This confirmed that m-learning is recognized as having potential for the learning process [85], for example for students’ voices, as one student confirmed. This confirms research pointing at m-learning as encouraging the student voice [86]. When modifying the m-learning materials and methods to meet the needs and learning preferences of their students, instructors also motivated their students’ engagement. Meta-cognitive strategies and immersion themes follow cognitive tactics; for meta-cognitive skills, students are aware of their learning process and have control over it since m-learning motivates them to learn flexibly and effortlessly, and the literature emphasizes this result [87,88]. The least mentioned theme for cognitive engagement is curiosity, though the literature emphasizes that this is due to the cognitive curiosity and interest that are present in certain apps such as games [89]. The current findings suggest that instructors can encourage cognitive curiosity and interest while being aware of the function of specific mobile activities, such as gamification tools. When describing emotional engagement, the students mentioned different emotions such as excitement and enjoyment. The participants enjoyed this new experience as holding mobile devices to access learning content on the Moodle application made them feel relaxed and happy. Students’ responses showed positive emotions, such as enjoyment toward m-learning, interactive activities, and new tools, which point to the emotional dimension of engagement [29]. Another aspect that inspires and supports students’ emotional engagement is when instructors offer them comfort; this perspective is consistent with Tang and Hew [90]. Describing behavioral engagement, the students mentioned different themes, such as attendance and effort on tasks, where students had grit in facing and solving problems while accessing the Moodle mobile application. This sub-theme implies that students devote time and effort to learning during class to acquire particular knowledge and skills. The outcomes are in line with those of other studies [91,92]. On the other hand, using the Moodle mobile application seems to have contributed to high engagement levels with different dimensions in terms of the total number of posts, comments, tasks, and student interactions on the course. Participation, task completion, and interaction rate have indicated students’ engagement.

Due to the interactive activities that give students more responsibility and control over their mobile social interactions, there is an increase in social engagement when using m-learning, as evidenced by the increased number of mobile social interaction indicators. Learners are connected continuously to what suits their needs and how they prefer to interact; therefore, m-learning empowers learners to boost the level of social interaction, which is different from other learning formats [84].

## 5. Research Conclusions

The current study results indicate that m-learning enriches students’ engagement. Based on the study’s findings, it is suggested that instructors should offer assistance, support, and encouragement to students using m-learning, as this can increase their confidence and keep them interested. This confidence and interest would motivate their affective engagement. As a result, the other types of their engagement would increase. Specifically, a confident student would become more involved in problem solving, and would try new methods to solve a problem. In addition, a confident student would not hesitate to be part of a group that tries to learn a topic or to solve problems related to the new topic.

Through inclusive and interactive nuances, students experienced an interactive environment, which helped to develop friendly and positive interpersonal relationships between participants. Software developers need to develop new applications for the mobile phone that are interactive tools, so that through the interactivity with the software, the student increases his or her social engagement, and as a result increases their cognitive engagement as well as their behavioral engagement.

Moreover, the findings suggest that the instructor, when focusing on creating interactive content that personalizes learning with various tools, can encourage different dimensions of engagement. Thus, instructors need to create interactive content that uses various tools.

In addition to the previously mentioned attributes, as a result of its interactivity, accessibility, and ease of use, m-learning motivated the students that participated in the research. Other environments are viable to encourage students’ engagement, as with online learning, but m-learning is distinguished from other learning environments in its accessibility and ease of use, in addition to its familiarity to the students, so it would encourage the engagement of students more than the other environments.

## 6. Research Limitations and Future Research

The study uses qualitative methods through interviews to investigate students’ engagement in the mobile environment. A recommendation for future research is to employ controlled studies that investigate student engagement by using learning analytics.

In addition to the above, as the present study follows qualitative methods, future quantitative research is needed to study students’ engagement in m-learning. Quantitative research could address background variables, such as gender, university year, and technological qualification, and whether they influence students’ engagement in mobile contexts.

The instructional design paradigm used in the course was ADDIE (analysis, design, development, implementation, and evaluation). More efforts need to be dedicated to the instructional design process for mobile activities. Various designs could be used to enhance the students’ learning experiences. Future research could examine the influence of design on students’ experiences, especially their engagement in the learning process.

Generalizability is one of the present study’s limitations. The study was conducted in a bachelor’s degree course. Designing mobile-based courses in different contexts, such as K-12 and post-graduate settings, is intriguing. Another limitation of the present study is that it examined students’ use of mobile devices for one semester (fourteen weeks). More longitudinal studies would shed light on the engagement phenomenon in mobile platforms.

In addition to the above, the first author is a lecturer for university students who took part in the academic course. To overcome this limitation, the researchers examined the trustworthiness of the data collection and analysis tools, as described in the methodology section. Despite this, future research needs to be conducted with people who are not the researcher’s students. Moreover, the interview questions did not address the shortcomings of m-learning regarding students’ engagement. Future research could address this issue by taking into consideration a theoretical framework such as the one the researchers utilized here.

## Figures and Tables

**Table 1:** Themes used from the theory and their codes.

Category	Themes	Codes
Social Engagement	Competition	Winning, losing, likes to be recognized.
	Building community	Sharing, working together, and participating in activity.
	Mobile social interaction	Interaction with content, interaction with interface, discussion, interaction with peers, interaction with instructor.
	Developing relationship	Connections, became friends outside class.
	Sense of belonging	Close relationship with peers, close relationship with teachers.
Cognitive Engagement	Attention	Attention, stay focused.
	Cognitive strategies	Remembering, memorizing, analyzing.
	Meta-cognitive strategies	Problem solving, evaluation, monitor their own progress, self-reflect.
	Immersion	Not feeling the time, state of flow.
	Cognitive curiosity	Asking questions, interest.
Emotional Engagement	Excitement and enjoyment	I feel great—happy, at ease, wonderful, and enthusiastic, I was waiting for this class.
	Instructor comforts students	It makes a difference, the researchers need encouragement and guidance, she was supporting us.
	Motivation	I was motivated, I open the app at home frequently.
	Emotional safety	Feel confident, express self, less shy.
Behavioral Engagement	Effort and time on task	Spending time to solve problems on their own.
	Participation	Participates in discussions.
	Attendance	Regular attendance.
	Code of conduct	Respect, privacy.

## Data Availability

Not applicable.

## References

[1] Daher W. (2014). Students’ Adoption of Social Networks as Environments for Learning and Teaching: The Case of the Facebook. Int. J. Emerg. Technol. Learn. (iJET).

[2] Daher W., Baya’A N. (2011). Characteristics of middle school students learning actions in outdoor mathematical activities with the cellular phone. Teach. Math. Its Appl. Int. J. IMA.

[3] Shaqour A., Daher W. (2010). Factors Influencing Students’ Use of Electronic Resources and their Opinions About this Use: The Case of Students at An-Najah National University. Int. J. Emerg. Technol. Learn. (iJET).

[4] Abuzant M., Ghanem M., Abd-Rabo A., Daher W. (2021). Quality of Using Google Classroom to Support the Learning Processes in the Automation and Programming Course. Int. J. Emerg. Technol. Learn. (iJET).

[5] Pham X.L., Chen G.D. (2018). PACARD: A New Interface to Increase Mobile Learning App Engagement, Distributed Through App Stores. J. Educ. Comput. Res..

[6] Goundar M.S., Kumar B.A. (2021). The use of mobile learning applications in higher education institutes. Educ. Inf. Technol..

[7] Daher W. (2009). Preservice teachers’ perceptions of applets for solving mathematical problems: Need, difficulties and functions. J. Educ. Technol. Soc..

[8] Zhou M., Li Z. (2018). Blended mobile learning in theatre arts classrooms in higher education. Innov. Educ. Teach. Int..

[9] Cebron N., Sorgo L., Cunja E.V. (2021). Between traditional and mobile language learning. Bulletin of the Transilvania University of Brasov.

[10] Snezhko Z., Babaskin D., Vanina E., Rogulin R., Egorova Z. (2022). Motivation for Mobile Learning: Teacher Engagement and Built-In Mechanisms. Int. J. Interact. Mob. Technol. (iJIM).

[11] Oliveira D.M.D., Pedro L., Santos C. (2021). The use of mobile applications in higher education classes: A comparative pilot study of the students’ perceptions and real usage. Smart Learn. Environ..

[12] Crompton H., Bernacki M., Greene J. (2020). Psychological foundations of emerging technologies for teaching and learning in higher education. Curr. Opin. Psychol..

[13] Diacopoulos M., Crompton C. (2020). Education, A systematic review of mobile learning in social studies. Comput. Educ..

[14] Neffati O.S., Setiawan R., Jayanthi P., Vanithamani S., Sharma D.K., Regin R., Mani D., Sengan S. (2021). An educational tool for enhanced mobile e-Learning for technical higher education using mobile devices for augmented reality. Microprocess. Microsyst..

[15] Dunn T., Kennedy M. (2019). Technology Enhanced Learning in higher education; motivations, engagement and academic achievement. Comput. Educ..

[16] Liu C., Correia A.-P. (2021). A Case Study of Learners’ Engagement in Mobile Learning Applications. Online Learn..

[17] Kahu E., Nelson K. (2018). Student engagement in the educational interface: Understanding the mechanisms of student success. High. Educ. Res. Dev..

[18] Bankmycell How Many Smartphones Are in the World?. https://www.bankmycell.com/blog/how-many-phones-are-in-the-world.

[19] Senaratne S.I., Samarasinghe S.M. (2019). Factors Affecting the Intention to Adopt M-Learning. Int. Bus. Res..

[20] Naciri A., Baba M.A., Achbani A., Kharbach A. (2020). Mobile Learning in Higher Education: Unavoidable Alternative during COVID-19. Aquademia Water Environ. Technol..

[21] Xie K., Heddy B.C., Vongkulluksn V.W. (2019). Examining engagement in context using experience-sampling method with mobile technology. Contemp. Educ. Psychol..

[22] Bitrián P., Buil I., Catalán S. (2021). Enhancing user engagement: The role of gamification in mobile apps. J. Bus. Res..

[23] Kim S., Baek T.H. (2018). Examining the antecedents and consequences of mobile app engagement. Telemat. Inform..

[24] Rosman M.R.M., Ismail M.N., Masrek M.N. (2021). How Engaging Are You? Empirical Evidence from Malaysian Research Universities. Int. J. Interact. Mob. Technol. (iJIM).

[25] Alazaza N.S. (2018). Understanding continuous intention usage of mobile services in the Palestinian Higher education institutions. Up J. Res. Stud..

[26] Bowden J.L.-H., Tickle L., Naumann K. (2019). The four pillars of tertiary student engagement and success: A holistic measurement approach. Stud. High. Educ..

[27] Marzouki Y., Aldossari F.S., Veltri G.A. (2021). Understanding the buffering effect of social media use on anxiety during the COVID-19 pandemic lockdown. Humanit. Soc. Sci. Commun..

[28] Kumar B.A., Chand S.S. (2018). Mobile learning adoption: A systematic review. Educ. Inf. Technol..

[29] Moya S., Camacho M. (2021). Identifying the key success factors for the adoption of mobile learning. Educ. Inf. Technol..

[30] Alkhalaf S., Amasha M., Al-Jarallah A. (2017). Using m-Learning as an Effective Device in Teaching and Learning in Higher Education in Saudi Arabia. Int. J. Inf. Educ. Technol..

[31] Traxler C. (2020). Learning with mobiles or “mobile learning”. The Oxford Handbook of Mobile Communication and Society.

[32] Voigt T., Matthee M. (2012). Tablets with Restricted Mobility: Investigating User Acceptance in a South African Mathematics Mobile Learning Project. mLearn.

[33] Sletten M., Montebello (2021). Secure Mobile Learning. Procedia Comput. Sci..

[34] Al-Emran M., Alkhoudary Y., Mezhuyev V., Al-Emran M. (2019). Students and Educators Attitudes towards the use of M-Learning: Gender and Smartphone Ownership Differences. Int. J. Interact. Mob. Technol..

[35] Botero G.G., Questier F., Cincinnato S., He T., Zhu C. (2018). Acceptance and usage of mobile assisted language learning by higher education students. J. Comput. High. Educ..

[36] Muir T., Milthorpe N., Stone C., Dyment J., Freeman E., Hopwood B. (2019). Chronicling engagement: Students’ experience of online learning over time. Distance Educ..

[37] Sholikah M., Harsono D. (2021). Enhancing Student Involvement Based on Adoption Mobile Learning Innovation as Interactive Multimedia. Int. J. Interact. Mob. Technol..

[38] Farrell O., Brunton J. (2020). A balancing act: A window into online student engagement experiences. Int. J. Educ. Technol. High. Educ..

[39] Qashou A. (2021). Influencing factors in M-learning adoption in higher education. Educ. Inf. Technol..

[40] Bond M., Bedenlier S. (2019). Facilitating Student Engagement Through Educational Technology: Towards a Conceptual Framework. J. Interact. Media Educ..

[41] Nichter S. (2021). Does Mode of Access Make a Difference? Mobile Learning and Online Student Engagement. Online Learn..

[42] Fredricks J.A., Blumenfeld P.C., Paris A.H. (2004). School engagement: Potential of the concept, state of the evidence. Rev. Educ. Res..

[43] Fredricks J.A., Filsecker M., Lawson M.A. (2016). Student engagement, context, and adjustment: Addressing definitional, measurement, and methodological issues. Learn. Instr..

[44] Redmond P., Heffernan A., Abawi L., Brown A., Henderson R. (2018). An Online Engagement Framework for Higher Education. Online Learn..

[45] Chiu T.K.F. (2021). Digital support for student engagement in blended learning based on self-determination theory. Comput. Hum. Behav..

[46] Calder N., Larkin K., Sinclair N. (2018). Mobile technologies: How might using mobile technologies reshape the learning and teaching of mathematics?. Using Mobile Technologies in the Teaching and Learning of Mathematics.

[47] Carpenter S. (2019). What does social engagement mean and what should art education do about it?. Stud. Art Educ..

[48] Henrie C.R., Halverson L.R., Graham C.R. (2015). Measuring student engagement in technology-mediated learning: A review. Comput. Educ..

[49] Shafie H., Majid F.A., Ismail I.S. (2019). Technological Pedagogical Content Knowledge (TPACK) in Teaching 21st Century Skills in the 21st Century Classroom. Asian J. Univ. Educ..

[50] Chung E., Subramaniam G., Dass L.C. (2020). Online Learning Readiness Among University Students in Malaysia Amidst COVID-19. Asian J. Univ. Educ..

[51] Cho M.-H., Castañeda D. (2019). Motivational and affective engagement in learning Spanish with a mobile application. System.

[52] Mohammadi M., Sarvestani M.S., Nouroozi S. (2020). Mobile Phone Use in Education and Learning by Faculty Members of Technical-Engineering Groups: Concurrent Mixed Methods Design. Frontiers in Education.

[53] Turgeman L., Smart O., Guy N. (2019). Unsupervised learning approach to estimating user engagement with mobile applications: A case study of The Weather Company (IBM). Expert Syst. Appl..

[54] Heflin H., Shewmaker J., Nguyen J. (2017). Impact of mobile technology on student attitudes, engagement, and learning. Comput. Educ..

[55] Kuh G.D., Cruce T.M., Shoup R., Kinzie J., Gonyea R.M. (2008). Unmasking the effects of student engagement on first-year college grades and persistence. J. High. Educ..

[56] Krause K., Coates H. (2008). Students’ engagement in first-year university. Assess. Eval. High. Educ..

[57] D’Errico F., Paciello M., Cerniglia L. (2016). When emotions enhance students’ engagement in e-learning processes. J. e-Learn. Knowl. Soc..

[58] McCormick K.M., Plucker J.A. (2013). Connecting Student Engagement to the Academic and Social Needs of Gifted and Talented Students. Brill.

[59] Oertel C., Castellano G., Chetouani M., Nasir J., Obaid M., Pelachaud C., Peters C. (2020). Engagement in human-agent interaction: An overview. Front. Robot. AI.

[60] Razzaque A. (2020). M-Learning Improves Knowledge Sharing Over e-Learning Platforms to Build Higher Education Students’ Social Capital. SAGE Open.

[61] Crompton H., Burke D. (2018). The use of mobile learning in higher education: A systematic review. Comput. Educ..

[62] Silvermann D. (2013). Doing Qualitative Research: A Practical Handbook.

[63] Creswell J.W., Clark V.L.P. (2017). Designing and Conducting Mixed Methods Research.

[64] Xerri D. (2018). The Use of Interviews and Focus Groups in Teacher Research. Clear. House: A J. Educ. Strat. Issues Ideas.

[65] Gundumogula M. (2020). Importance of focus groups in qualitative research. Int. J. Humanit. Soc. Sci. (IJHSS).

[66] Krueger R.A., Casey M.A. (2000). Overview of focus groups. Focus Groups: A Pract. Guide Appl. Res..

[67] Harrell M.C., Bradley M.A. (2009). Data Collection Methods. Semi-Structured Interviews and Focus Groups.

[68] Cooksey R., McDonald G. (2019). Surviving and Thriving in Postgraduate Research.

[69] Cilesiz S. (2010). A phenomenological approach to experiences with technology: Current state, promise, and future directions for research. Educ. Technol. Res. Dev..

[70] Fielding N.G., Blank G., Lee R.M. (2008). The Sage Handbook of Online Research Methods.

[71] Creswell J.A. (1998). Five qualitative traditions of inquiry. Qualitative Inquiry and Research Design. Choosing among Five Traditions.

[72] Lambert S.D., Loiselle C. (2008). Combining individual interviews and focus groups to enhance data richness. J. Adv. Nurs..

[73] Singh Y.K. (2006). Fundamental of Research Methodology and Statistics.

[74] Ngozwana N. (2018). Ethical dilemmas in qualitative research methodology: Researcher’s reflections. Int. J. Educ. Methodol..

[75] Kyngäs H. (2020). Inductive content analysis. The Application of Content Analysis in Nursing Science Research.

[76] Daher W., Anabousy A., Alfahel E. (2022). Elementary teachers’ development in using technological tools to engage students in online learning. Eur. J. Ed. Res..

[77] Daher W., Sabbah K., Abuzant M. (2021). Affective engagement of higher education students in an online course. Emerg. Sci. J..

[78] Ricci F., Modenese A., Gobba F., Morlini I. (2022). Evaluation of an Online Course Promoting Health and Wellbeing for University Students and Employees. Eur. J. Investig. Health Psychol. Educ..

[79] Daher W., Salameh H. (2022). The Role of a Ministry of Education in Addressing Distance Education during Emergency Education. Eur. J. Investig. Health Psychol. Educ..

[80] Romano L., Angelini G., Consiglio P., Fiorilli C. (2021). Academic Resilience and Engagement in High School Students: The Mediating Role of Perceived Teacher Emotional Support. Eur. J. Investig. Health Psychol. Educ..

[81] Miller B., Thomas M. (2021). Accessing Learning Management Systems with Smartphones: What is the Effect on Learning Behavior and Student Engagement?. eLearning Engagement in a Transformative Social Learning Environment.

[82] Purwanti Y., Imania K.A.N., Rahadian D., Bariah S.H., Muljanto S. (2019). Mobile learning in promoting student’s engagement. J. Phys. Conf. Ser..

[83] Nugroho N. (2022). Students’ engagement in online learning using whatsapp group during COVID-19 pandemic. Linguistics ELT J..

[84] Tu C.-H., Sujo-Montes L.E. (2015). Mobile Learning and Mobile Social Interaction. Media Rich Instruction.

[85] Al-Razgan M., Alotaibi H. (2019). Personalized Mobile Learning System to Enhance Language Learning Outcomes. Indian J. Sci. Technol..

[86] Daher W. (2017). Student voice in the mobile phone environment: A grounded theory approach. Int. J. Mob. Blended Learn. (IJMBL).

[87] Daher W., Nimer B.A., Jaber O., Anabousy A. (2018). Developing pre-service mathematics teachers’ metacognitive thinking for learning and teaching with mobile technology. Eurasia Proc. Educ. Soc. Sci..

[88] Damopolii I., Kurniadi B. (2019). Training students metacognitive skill using mobile learning. J. Physics: Conf. Ser..

[89] Hochberg K., Kuhn J., Müller A. (2018). Using smartphones as experimental tools—Effects on interest, curiosity, and learning in physics education. J. Sci. Educ. Technol..

[90] Tang Y., Hew K.F. (2022). Effects of using mobile instant messaging on student behavioral, emotional, and cognitive engagement: A quasi-experimental study. Int. J. Educ. Technol. High. Educ..

[91] Bere A., Rambe P. (2019). Understanding mobile learning using a social embeddedness approach: A case of instant messaging. Int. J. Educ. Dev. Using Inf. Commun. Technol..

[92] Muhammet B., Okan S. (2018). Determining the readiness levels of pre-service teachers towards mobile learning in classroom management. Educ. Res. Rev..

